# What Do Medical Students Think About Incorporating VR Into Psychiatry Education and Training?

**DOI:** 10.1192/bjo.2022.153

**Published:** 2022-06-20

**Authors:** Manu Sidhu, Vikram Dulai

**Affiliations:** Imperial College School of Medicine, London, United Kingdom

## Abstract

**Aims:**

The authors hypothesised that medical students may be receptive to incorporating Virtual Reality (VR) within the psychiatric curriculum as a technological adjunct to existing teaching methods. Therefore, the aim was to evaluate medical students’ attitudes towards the role of VR in psychiatry education and training.

**Methods:**

In this descriptive cross-sectional online survey, 76 medical students from all year groups across 10 medical schools in the UK answered a questionnaire consisting of 13 items regarding their views on the role of VR in psychiatry education and training, each on a 5-point Likert scale.

**Results:**

96.1% of respondents had received some level of education and training in psychiatry. 57.9% had never undertaken a VR experience before, yet 79.0% ‘agreed/strongly agreed’ that they would feel comfortable using VR at medical school.

71.1% ‘agreed/strongly agreed’ that experiencing the first-person perspectives of psychiatric patients in VR would enable them to develop greater empathy. 81.6% ‘agreed/strongly agreed’ that managing dangerous patient interactions in VR would increase their confidence in managing such interactions in real-world clinical settings. However, students were most ‘unsure’ about whether VR would reduce their anxiety (30.3%) and improve their interpersonal communication (27.6%) in real-world clinical settings.

The majority of students ‘agreed/strongly agreed’ that VR would make educational experiences more engaging (80.3%), improve understanding of content (63.1%), and better prepare them for clinical practice (64.4%).

Most significantly, 81.6% ‘agreed/strongly agreed’ that learning in VR would enhance experiential learning in ways that existing teaching methods outside of clinical settings cannot, and 84.2% ‘agreed/strongly agreed’ that they would rather learn from a mixture of VR plus existing methods over existing methods alone.

**Conclusion:**

These findings demonstrate that medical students believe VR would improve engagement, understanding, and preparation for clinical practice.

VR holds the greatest potential in developing empathy for patients with mental illness and preparing students for dangerous patient interactions. However, further investigation is required to evaluate how educational experiences in VR translate to performance in real-world clinical settings.

In times of restricted access to clinical care, such as during the COVID-19 pandemic, VR could play a vital role in teaching psychiatry. The preference for VR to be added to existing teaching methods was the strongest held and most relevant belief to the aim of this study, indicating the readiness of medical students to accept VR into psychiatry education and training.

